# Interpersonal Coordination: Methods, Achievements, and Challenges

**DOI:** 10.3389/fpsyg.2017.01685

**Published:** 2017-09-27

**Authors:** Carlos Cornejo, Zamara Cuadros, Ricardo Morales, Javiera Paredes

**Affiliations:** Laboratorio de Lenguaje Interacción y Fenomenología, Escuela de Psicología, Pontificia Universidad Católica de Chile, Santiago, Chile

**Keywords:** interpersonal coordination, interactional synchrony, video analysis, microanalysis, motion capture, hyperscanning

## Abstract

Research regarding interpersonal coordination can be traced back to the early 1960s when video recording began to be utilized in communication studies. Since then, technological advances have extended the range of techniques that can be used to accurately study interactional phenomena. Although such a diversity of methods contributes to the improvement of knowledge concerning interpersonal coordination, it has become increasingly difficult to maintain a comprehensive view of the field. In the present article, we review the main capture methods by describing their major findings, levels of description and limitations. We group them into three categories: video analysis, motion tracking, and psychophysiological and neurophysiological techniques. Revised evidence suggests that interpersonal coordination encompasses a family of morphological and temporal synchronies at different levels and that it is closely related to the construction and maintenance of a common social and affective space. We conclude by arguing that future research should address methodological challenges to advance the understanding of coordination phenomena.

## Introduction

Studies from different fields have demonstrated that human beings spontaneously display behavioral, gestural and linguistic coordination during interactions with peers. These coordinative patterns have been variously termed alignment (Garrod and Anderson, 1987; Garrod and Pickering, 2004), behavioral matching (Bernieri and Rosenthal, 1991; Louwerse et al., 2012), mimicry (Lakin et al., 2008; Cheung et al., 2015) and interactional synchrony (Fine et al., 2013; Endedijk et al., 2015). In general, interpersonal coordination phenomena have been understood as spontaneous temporal synchronization of body movements and/or linguistic utterances between people when they engage in a social interaction (Bernieri et al., 1988).

Most knowledge regarding the factors and consequences of interpersonal coordination has been produced recently as technological advances have made it possible to describe synchronized body movements accurately and in detail. Currently, there are various methods to capture interpersonal coordination, ranging from microanalytical video processing to neuro-hyperscanning. Although this diversity of methods certainly contributes to the acquisition of more complete knowledge regarding interpersonal coordination, it has become increasingly difficult to maintain a comprehensive view of the field. The more specialized technical devices become, the more specific their description of coordination patterns is. Therefore, the expansion of methods may lead researchers to overlook connections among coordination phenomena described at different levels by means of different technologies.

Recent publications have provided overviews of empirical findings (Rennung and Göritz, 2016; Vicaria and Dickens, 2016) as well as methods for capturing and analyzing patterns of coordinated movement (Rein, 2016; Chetouani et al., 2017; Jakubowski et al., 2017). For example, Vicaria and Dickens (2016) presented an exhaustive meta-analysis of interpersonal coordination outcomes, and Rennung and Göritz (2016) did the same with the prosocial consequences of interpersonal synchrony. Other recent publications reviewed findings of interpersonal coordination in particular samples, such as patients with mental disorders (Del-Monte et al., 2013), and specific contexts such as dance (Torrents et al., 2016) and sports (Araújo et al., 2016; Passos and Chow, 2016; Yamamoto et al., 2016; Seifert et al., 2017). Jakubowski et al. (2017) compared the efficacy of three different computer vision techniques with motion capture systems to study interpersonal coordination in musical performances. Rein (2016) presented a meticulous review of modern analysis techniques to measure interpersonal coordination. Though relevant, such efforts have, until now, focused on particular levels, outcomes or techniques related to interpersonal coordination and have been of limited use for advancing an overall understanding of the phenomenon. In the present article, we present a wider review of the main methods by which interpersonal coordination has been studied and the major findings at different levels of observation.

Our starting point is the classical definition of interpersonal coordination (henceforth: IC) by Bernieri and Rosenthal (1991, p. 403): “the degree to which the behaviors in an interaction are non-random, patterned or synchronized in both timing [and] form.” According to this general definition, interpersonal coordination should satisfy the following defining attributes: (a) it is a social phenomenon involving two or more persons (Bernieri et al., 1988; Schmidt and Richardson, 2008; Marsh et al., 2009); (b) it occurs during simultaneous face-to-face interaction (i.e., it requires the real co-presence of another person) (Bernieri and Rosenthal, 1991); and (c) it emerges rapidly (in a short time frame) and spontaneously (without conscious control or intentionality) (Issartel et al., 2007; van Ulzen et al., 2008; Miles et al., 2010; Coey et al., 2011; Demos et al., 2012; Davis, 2016). According to this definition, we excluded from this review those studies that did not satisfy the abovementioned attributes. For example, we did not examine studies on intrapersonal coordination (Jung et al., 2011; Ramenzoni et al., 2011, 2012) because they disregarded the social nature of interpersonal coordination. In some studies, the requirement for the real-time co-presence of people was overlooked. Therefore, we included all studies in which one participant observed previously recorded video or audio of another participant and believed that he/she was interacting online with a real person.

Below, we describe the methods for capturing interpersonal coordination and indicate their features, advantages, and disadvantages as well as selected examples. These methods will be presented in three main classes: video, motion tracking, and physiological measures. We conclude by discussing the current methodological challenges of measuring real-life interpersonal coordination.

## Video Analysis

Video recording was the first method utilized to study interpersonal coordination (Condon and Ogston, 1966; Condon and Sander, 1974). This method allowed researchers to record and quantify movement in a degree of detail never seen before. Recently, it has become an accessible and easy way to measure synchrony because video data are now digital and analysis is automated. Contingent on the technique utilized to analyze video data, four main categories of video studies are recognized: microanalysis (Condon and Ogston, 1966; Kendon, 1970; Condon and Sander, 1974), behavioral coding (Bernieri, 1988; Feldman, 2003; Nagaoka et al., 2006), motion energy analysis (Grammer et al., 1999; Ramseyer and Tschacher, 2011; Paxton and Dale, 2013a,b,c), and digital plotting of movement (Passos et al., 2008; Bourbousson et al., 2010).

### Microanalysis

Microanalysis is an analysis procedure pioneered by Birdwhistell (1952), Scheflen (1964), and Condon and Ogston (1966) that adapts cinematographic techniques to study human behavior. For example, Condon and Ogston (1966) examined the movement regularities of people in communicative contexts. They analyzed segments of video recordings by means of equipment that allowed them to slow the film and examine intervals as small as 1/48 s. Condon and Ogston (1966) codified visible movements of participants’ body parts, such as the head, eyes, feet and even fingers. Investigators analyzed movements frame by frame, identifying and coding their trajectory through a selected film segment. This led them to recognize regular micropatterns of communicational behavior not only at an intra-individual level but also at an inter-individual level. In a later study, Condon and Sander (1974) analyzed adult-neonate dyads and searched for coordination patterns between adults’ speech and neonates’ movements. Condon and Sander codified synchrony through “process units” of movement such as flexion, inclination and rotation of parts of the participant’s body, such as the head, eyes and wrists. An hour of segments from different dyads was analyzed. These segments ranged from a few sentences spoken by the adult to long utterances shared by the infant and adult. The researchers discovered that neonates synchronized their bodily movements with adult speech as early as the 1st day of life. Interestingly, this coordination did not occur when neonates were exposed to isolated vowel sounds. According to the researchers, this finding suggested that “…the ‘bond’ between human beings [was] an expression of participation within shared organizational forms rather than as isolated entities sending discrete messages” (Condon and Sander, 1974, p. 462). Another study conducted by Kendon (1970) focused on movements of participants in a conversation compared with movements of people who were merely listening to the same exchange. He analyzed an 8-min video recording of a group of people talking in a hotel lobby. Movements of participants were coded frame by frame in selected film segments of approximately 6 s in a protocol similar to that of Condon and Ogston (1966). Kendon found very precise temporal synchrony among participants even if they were not directly looking at each other or actively participating in the conversation. The author suggested that interpersonal coordination played a crucial role “since it provides one of the ways in which two people signal that they are ‘open’ to one another, and not to others” (Kendon, 1970, p. 124). A more recent study using microanalysis explored movement patterns and coordinative behavior of therapist and patient in a dance therapy context (Houghton and Beebe, 2016).

### Behavioral Coding

Behavioral coding tracks global behaviors – rather than specific movements – as an index of interpersonal coordination. This procedure typically relates interpersonal coordination to other psychological variables (LaFrance, 1977; Bernieri et al., 1988; Chartrand and Bargh, 1999). For example, Bernieri (1988) studied the relationship between interpersonal coordination and rapport. Dyads of high school students were videotaped for 10 min teaching each other a simple task. With Likert scales, Bernieri (1988) measured four variables: simultaneous movement, tempo similarity, coordination and dance like smoothness, and behavior matching. Interpersonal coordination was measured according to the degree of perceived movement synchrony and behavioral matching that participants displayed in the recordings. Interestingly, Bernieri (1988) found that the degree of interpersonal coordination rated by judges was strongly related to self-reported rapport (Bernieri, 1988). In another example of behavioral coding, Chartrand and Bargh (1999) reported unintentional mirroring in interactional settings, which they called the “Chameleon Effect.” In three experimental studies, dyads were asked to describe a set of photographs. Each dyad was composed of a participant and a partner. Mirroring was coded by identifying three key behaviors throughout the interaction: participants’ smiles, participants’ rubbing of their own face and the shaking of their feet. The first study showed that participants unintentionally synchronized with their partner while participating in the task. The second experiment manipulated the degree to which the partner mimicked the participant during the task. When the decree of mimicry increased, participants reported an increased liking for their task partner, and the analysis suggested that mimicry facilitated smoothness of the interaction. Finally, the third experiment showed that participants who scored higher on an empathy test matched their partner’s behavior more. Other studies using this type of analysis have explored the co-regulation of arousal in dyads of parents and children (Feldman, 2003), infant helping behaviors (Cirelli et al., 2017), interpersonal synchrony and its relationship with self-reports of rapport in adults (LaFrance, 1977), the mutuality of mother–infant interaction (Tronick et al., 1977), the relationship between synchrony and psychotherapeutic outcomes (Nagaoka et al., 2006), the relationship between parent–child synchronization and the diagnosis of Rett Syndrome/Autism (Trevarthen and Daniel, 2005) and the effect of practice and pressure in failures of interpersonal coordination (Ogawa and Sekiya, 2016).

### Motion Energy Analysis

Motion energy analysis (MEA) consists of an automated analysis of video-recorded movements. MEA calculates the difference in grayscale between two frames to measure the total amount of pixel change. The amount of pixel change signals the quantity of movement of each limb or individual selected for analysis. This value is filtered to reduce noise and flashes due to low recording quality or instability of the electrical installation of the experimental setting (Grammer et al., 1999; Kupper et al., 2010). Analysis is performed in predefined areas of interest from the selected video frame. A study using MEA was conducted by Ramseyer and Tschacher (2011) to explore interpersonal coordination as a reflection of therapist-patient relationship quality and therapy outcome. Two cameras were installed in the therapist’s office, and records were combined into a split-screen image to enable offline analysis. Once the session was recorded, coordination between participants was analyzed by MEA designating the whole body as an area of interest (ROI). The results showed that higher interpersonal synchrony was a characteristic of therapeutic processes that achieved higher symptom reduction. In addition, higher synchrony with the therapist was positively correlated with the patient’s rating of the relationship.

Paxton and Dale (2013b) worked with a variation of this analysis, namely, the “frame differencing method” (FDM). FDM differs from MEA in that it allows researchers to utilize a simplified script in pre-existing software, such as MATLAB, to perform the analysis. Its output provides an overall measure of body movement. An example of this technique can be found in a study involving dyads in affiliative and argumentative conversations (Paxton and Dale, 2013a). In this study, original images from video data were halved to separate participants in the conversation and then analyzed with FDM. The findings showed that interpersonal synchrony occurred only in dyads that participated in the affiliative condition in which participants discussed topics on which they agreed. Conversely, lower coordination was found in the argumentative condition in which participants discussed topics on which they disagreed and had to convince the other to change his/her mind (Paxton and Dale, 2013a). Other studies using FDM have explored synchrony in dyads in cooperation tasks (Abney et al., 2015) and the effects of body-oriented psychotherapy in schizophrenia patients (Galbusera et al., 2016). An alternative utilization of this method combines FDM with cross-spectral coherence analysis (CSC) (Allsop et al., 2016; Fujiwara and Daibo, 2016), which calculates the association between two time series as a function of frequency (Coey et al., 2011).

Another development that analyzes motion energy in video recording is correlation map analysis (CMA) (Latif et al., 2014). This analysis incorporates a Horn and Schunck algorithm in the video analysis. According to Latif et al. (2014), CMA allows a more sensitive measure of movement than FDM or earlier MEAs. Latif et al. (2014) studied interpersonal coordination in dyads that were friends or strangers and the way this distinction affected the perception of an external observer regarding the level of affiliation of the dyad. CMA was utilized to compute measures of movement and coordination of the dyads in a broad region of interest that encompassed the entire bodies of both participants. Friend dyads coordinated more frequently than stranger dyads did. It was additionally reported that an observer judged affiliation between dyads when information regarding the dyad was not restricted to the head and face regions (Latif et al., 2014).

### Digital Movement Plotting

Video recordings of sports activities have been analyzed by a digital plotting technique in search of interactional synchrony. Typically, the aim of these studies is to find interaction patterns in sports activities according to dynamical system modeling because such patterns have been found in other domains of human activity (Schmidt and O’Brien, 1997; Richardson et al., 2005). Video cameras are utilized to record players’ movements on a playing field. Then, video files are digitized by specialized software that allows the bi-dimensional coordinates of each participant to be plotted. This procedure is conducted by establishing two vectors located within the image of the body from an abdominal central point in each frame of the recording. The result is positional data of players’ displacement trajectories that allow the spatial coordinates of a dyad to be extracted using direct linear transformation. Finally, data are filtered at different frequencies in search of coordination patterns among the players (Passos et al., 2008; Bourbousson et al., 2010; Duarte et al., 2012). This technique was employed in a study conducted by Duarte et al. (2012) that explored the influence of interpersonal coordination on performance in soccer forward-defender dyads. The results of the digital plotting analysis showed that a forward’s success was based largely on spatiotemporal synchronization and unpredictability in interpersonal coordination processes, whereas a defender’s success was explained by his/her capacity to lead the interaction and a more predictable interpersonal coordination mode between the dyad. Similar studies have been conducted exploring the dynamics of tennis players as well as forward-defender dynamics in rugby, basketball and soccer (Palut and Zanone, 2005; Bourbousson et al., 2010; Esteves et al., 2012; Correia et al., 2016; Passos et al., 2017).

More recently, the automated digital plotting technique has been utilized for the study of finer forms of interpersonal coordination. Kodama et al. (2015) measured the intrapersonal and interpersonal coordination of participants’ fingers during a tapping task to explore the dynamic patterns in human movement described by Kelso (1984). Individual participants and dyads participated in intra- and interpersonal studies, respectively. The task consisted of tapping in phase (two fingers tapping in synchrony) or anti-phase (two fingers tapping alternatively) modes for 30 s according to a rhythm given by a metronome. The camera captured finger movement through automatic detection of the bi-dimensional motion of the fingers. Non-linear analyses for two time series and cross-recurrence quantification analyses were conducted to measure coordination. The results showed that overall movement stability was higher in the intrapersonal than in the interpersonal condition. In the interpersonal condition, there was no significant difference between the phase and anti-phase modes in movement stability.

## Motion Tracking Methods

Motion tracking systems have been utilized as an alternative method to track body movements during human interactions. This type of system automates data collection by tracking small markers within a one-, two-, or three-dimensional space. Markers are attached either to specific body parts of a person or to an object manipulated by a person. This method implies advantages for researchers, such as saving time in video processing and removing the necessity of researcher interaction with raw data (Paxton and Dale, 2013b). The most frequently utilized types of these methods are accelerometers, potentiometers, electrogoniometers, magnetic and optical motion capture systems.

### Accelerometers

Accelerometers can be attached to the human body, enabling the measurement of motion acceleration in one, two, or three dimensions (Troiano et al., 2014). They are additionally useful to obtain measures related to acceleration such as speed, vibration deviation and intensity of movements. Currently, accelerometers take the form of small sensor chips that can be used independently or through multiple portable everyday objects that incorporate them, such as watches and mobile phones. These devices have become a flexible, economical, comfortable and alternative way to track the body movements of two or more people engaged in a social interaction. Despite the real possibility of being used simultaneously to study multiple factors impacting body coordination, accelerometers have typically been utilized to inquire into musical features that could affect the emergence of coordinated interpersonal movement patterns in natural and semi-natural contexts. Accelerometers have recently been utilized to study the coordination of people dancing together to the rhythm of music (Ellamil et al., 2016; Lang et al., 2016; Tarr et al., 2016) or to the rhythm of a metronome (Brown and Meulenbroek, 2016). For example, Ellamil et al. (2016) investigated the influence of various musical features on group synchrony. Researchers positioned pairs of smartphones at waist level to obtain measures of the linear and three-dimensional acceleration of movements of people who danced together in groups of six in a dance club. In this study, group synchrony was analyzed by the estimation of instantaneous phase synchronization and intersubject phase synchronization as well as a cluster-phase method. Ellamil et al. (2016) found synchrony between participants’ forward and backward torso movements and found that such group synchrony was associated with musical track popularity and with musical pulsations similar to a walking pace. In addition, accelerometer sensors placed on participants’ wrists were utilized in a study conducted by Lang et al. (2016) that aimed to explore whether exposure to musical rhythm could increase coordination of movements. Acceleration measures of hand movements from dyads that jointly performed a labyrinth task while hearing one of three auditory stimuli (white noise, arrhythmic or rhythmic sound) were analyzed through cross-recurrence quantification analysis (CRQA). This method enables the configuration of dyad coupling patterns to be identified and quantified across time (i.e., it favors the comparison of two time series that co-evolve over time) (Coco and Dale, 2013; Fusaroli et al., 2014; Gates and Liu, 2016). The results revealed that exposure to musical rhythm enhanced interpersonal motor coupling. Recently, accelerometers have been utilized as part of a system called Powerline that is composed of multiple motion capture instruments. This system was developed to simultaneously obtain measurements of the angles and forces of rowing team members and the acceleration and speed of boats. For example, the utilization of this system combined with phenomenological measures allowed Seifert et al. (2017) to study the interpersonal coordination of two pairs of rowers who participated in a race against the clock. Researchers found evidence of complex dynamic interpersonal coordination among rower pairs that was adapted according to its effect on performance. Therefore, Seifert et al. (2017) emphasized that the utilization of different data sources enriched the understanding of complexities underlying the interpersonal coordination of rower pairs.

### Potentiometers

Potentiometers are instruments that measure electrical potential differences. When motion occurs, an electric afferent of the potentiometer transmits a continuous record of the angle adopted by objects moved by a person during a specific time series (Kautzor, 2015). Therefore, these devices enable an indirect measure of body movement. A potentiometer attached to a rigid pendulum that can only be moved back and forth in a single direction has been used to measure the spatial displacement of the hand that moves it in tasks that require coordination of movement between two people sitting side by side in chairs (Marmelat and Delignières, 2012; Del-Monte et al., 2013; Varlet et al., 2014a; Ouwehand and Peper, 2015; Benerink et al., 2016). As with most time series data, angular displacement data recorded by potentiometers require processing and analysis that include at least three components: first, the application of filters to reduce noise from movements, such as a bidirectional Butterworth filter; second, the estimation of the relative phase between angular displacements of participants’ pendulums, typically by using a Hilbert transform; third, the computation of coordination variability and phase shift to analyze synchronization stability and establish which participant followed or led coordination with his/her partner. In studies using potentiometers, synchronization stability has traditionally been calculated through cross-correlations or non-linear analysis methods, such as circular variance of the relative phase (CVRP). In different ways, both of these enable estimates of covariation across time of angular displacement values in a two-dimensional plane for two individuals, yielding a coordination index that ranges from 0 (no coordination) to 1 (absolute coordination). An alternative and less frequently utilized method to measure synchronization stability is the calculation of standard deviation of the relative phase (SDRP). For example, by means of a computer-monitored potentiometer attached to each pendulum, Del-Monte et al. (2013) accurately measured angular displacements of hand movements and examined the interpersonal motor coordination of unaffected first-degree relatives of schizophrenia patients while synchronizing the swinging of the pendulum with healthy controls. Del-Monte et al. (2013) instructed dyads to oscillate pendulums at their own tempo and to maintain them during three segments of 30 s each. In the first and third segments, participants could see the movements of their partners, whereas in the second one, they could not see them. Then, researchers asked dyads to deliberately coordinate the swinging of their pendulums in an in-phase or anti-phase mode for 60 s. Variability of coordination among participants during intentional and unintentional trials was calculated by means of CVRP. Interestingly, the results showed that relatives of patients had greater variability of coordination than controls did and that they did not lead the coordination in the condition where they were explicitly asked to synchronize with their interaction partners. Consequently, the researchers concluded that although relatives of patients spontaneously coordinated their hand movements with those of their interaction partners, they had difficulties intentionally synchronizing with them. These results and conclusions are concordant with the report of Varlet et al. (2014a) regarding patterns of social motor coordination between patients with social anxiety disorder and healthy controls performing pendulum coordination tasks. Varlet et al. (2014a) employed a design and procedure similar to that of Del-Monte et al. (2013); however, they analyzed synchronization stability differently. Through the calculation of SDRP for intentional coordination situations and CVRP for unintentional conditions, Varlet et al. (2014a) determined that patients had lower stability in the intentional coordination of their movements with their interaction partners, whereas their unintentional coordination remained similar to that of the control group. These findings showed the usefulness of potentiometers to detect motor patterns that could be utilized as a trait marker of specific mental and social disorders. Other studies using this type of capture and analysis method have explored global patterns of anticipation between members of dyads (Marmelat and Delignières, 2012) and examined differences between interpersonal movement synchronization and human-object synchronization (Ouwehand and Peper, 2015).

### Electrogoniometers

These instruments are electronic devices that directly measure angles of uniplanar movements produced by specific limbs of a person in a time series (Shiratsu and Coury, 2003; Wang et al., 2011). Such devices attached to the wrist, hands or elbow have been utilized to record arm movements in arm-curl tasks (Miles et al., 2011; Lumsden et al., 2012) and pendulum coordination tasks (Coey et al., 2011) performed individually or jointly. Data captured by means of electrogoniometers have been analyzed using the same three phases mentioned above for potentiometer data. However, non-linear analytical methods utilized in the third phase differ. In particular, the stability of interpersonal synchrony has been calculated by one or both of two measures: (CSC) and the distribution of relative phase angles (DRP). The first measure estimates the degree of association between a two-movement time series as a function of frequency peaks, so a correlation value of 0 represents no coordination, whereas a value of 1 means absolute coordination (Coey et al., 2011). The second measure assesses interpersonal coordination by calculating the frequency of synchrony between the movements of two individuals within each of nine ranges of relative phase (20° each) (Coey et al., 2011; Miles et al., 2011). Consequently, “[c]oordination is indicated by concentration of relative phase angles in the regions of distribution near 0° (i.e., in-phase coordination) and/or 180° (i.e., anti-phase coordination)” (Miles et al., 2011, p. 498). These devices and analysis techniques have been employed mostly to explore factors that influence the spontaneous emergence of interpersonal synchrony. For instance, a study conducted by Coey et al. (2011) explored bidirectional influences between the emergence of spontaneous interpersonal coordination and the stability of intrapersonal interlimb coordination by attaching one electrogoniometer to each wrist of each participant and performing both CSC and DRP analyses. In this study, dyads sitting side by side participated in joint resolution of a puzzle task while simultaneously performing individual pendulum coordination. Specific analysis of four 2-min trials of in-phase and anti-phase pendulum oscillation in conditions in which participants could or could not see each other revealed that the emergence of interpersonal coordination was not influenced by the stability of intrapersonal coordination but by the visual perception of the partner’s movements. Therefore, the researchers suggested that spontaneous interpersonal coordination and the stability of intrapersonal movement patterns are not dependent on one another. In contrast, other studies that utilized electrogoniometers to record movements of both arms of each participant during an arm-curl task performed with a metronome and a virtual partner found that the emergence of interpersonal coordination was impacted by factors such as group membership (Miles et al., 2011) and social motives (Lumsden et al., 2012).

### Magnetic Motion Capture Systems

Operating with a different technology from the abovementioned systems, magnetic motion tracking devices simultaneously measure the positions of multiple magnetic sensors that can be attached to objects manipulated by people (Richardson et al., 2005, 2007; Demos et al., 2012; Lumsden et al., 2012; Washburn et al., 2014; Fitzpatrick et al., 2016) or to different limbs of the human body (Ramenzoni et al., 2012; Athreya et al., 2014; Tolston et al., 2014). Through these systems, it is possible to obtain both indirect and direct measures of body movements. Most studies using magnetic sensors apply the same analysis method employed in studies with potentiometers and electrogoniometers. However, in addition to cross-correlations, CVRP, SDRP, CSC, and DRP, analysis of interpersonal synchrony stability includes CRQA. Magnetic motion capture systems have been utilized in multiple studies investigating perceptual and social factors that affect the spontaneous emergence of coordinated motion patterns. For instance, Tolston et al. (2014) utilized three magnetic sensors attached to the waist, hand, and head of each participant to explore whether constraining movement during joint tasks affected interpersonal coordination. Researchers asked dyads to talk to solve a picture-puzzle task in three conditions: both participants’ hands were restrained, both participants’ hands were free, or the hands of one participant were restrained while the hands of the other participant were free. Then, CRQA was executed to measure interpersonal coordination. The results demonstrated that postural interpersonal coordination declined when movement was constrained, especially when the hand movements of one participant were restrained. In another study, Demos et al. (2012) explored the influence of visual and auditory information regarding interpersonal synchrony by attaching one magnetic sensor to the chair headrest of each participant, as previously performed by Richardson et al. (2005, 2007). In this study, pairs of participants seated side by side in rocking chairs (positioned 0.5 m apart) participated in six trials consisting of a combination of visual (vision, no vision) and auditory (no sound, rocking sound, music) conditions. Through cross-correlation analysis, the researchers found that spontaneous coordination occurred under the conditions in which participants could see or hear each other. In another study (Athreya et al., 2014), two magnetic sensors attached to each participant’s right index fingertip and lumbar torso were employed to explore the influence of visual information on postural and manual interpersonal coordination. Participant dyads were instructed to coordinate their finger movements under conditions in which only the partner’s fingertip movements could be seen through laser pointer dots projected onto a black screen placed between them or conditions in which the partner’s whole-body movements could be seen in the absence of any screen. Dyads participated in six trials, each lasting 60 s, and data were analyzed by CRQA. The researchers confirmed previous findings that highlight the role of vision in interpersonal coordination; fingertip and postural interpersonal coordination were enhanced by visual information regarding the partner’s body movements. In addition, Miles et al. (2010) studied the link between social factors and behavioral matching by attaching two magnetic sensors to the lateral part of each leg above the knee. Participants participated in a stepping task with a female partner, who arrived 15 min late for half of the subjects. Analysis of coordination stability in each partner-participant dyad was conducted by means of DRP. The results indicated that in-phase synchrony was significantly reduced when participants interacted with a tardy partner. Other studies using magnetic motion capture systems reported that unintentional interpersonal coordination was enhanced by joint performance compared with solo performance (Ramenzoni et al., 2012) and by joint interaction with a prosocial partner in contrast to a pro-self partner (Lumsden et al., 2012).

### Optical Motion Capture Systems

These systems are particularly suitable for studying the coordinated movements of people in joint tasks. Optical motion capture allows body movements to be tracked using high-resolution infrared cameras to capture sensors that can be attached directly to body limbs or on special suits. Infrared cameras capture the position of each infrared reflective marker placed on the body with a specific sampling frequency. One of the advantages of optical motion capture systems is that they can be utilized to accurately track the free movement of more than one limb. However, these systems have mainly been utilized with a few markers for tracking the coordination of specific body parts, such as hand positions of dyads (Gueugnon et al., 2016; Gorman et al., 2017), the position of peoples’ heads during joint tasks (Kijima et al., 2017), coordination of finger motion between dyads (Oullier et al., 2008; Fine et al., 2015), coordination of foot movements between dyads (Vesper et al., 2013) and coordination of heads and hands between dyads (Dammeyer and Køppe, 2013). To a lesser extent, optical motion capture systems have been utilized for tracking upper body movements by placing more than one or two infrared sensors on people performing joint tasks (Varlet et al., 2011; Llobera et al., 2016; Preissmann et al., 2016; Stevanovic et al., 2017), playing music together (Ragert et al., 2013; Glowinski et al., 2015; Jakubowski et al., 2017) or moving together to the rhythm of music (Burger et al., 2014; Toiviainen et al., 2014). Analysis of interpersonal coordination has been mainly performed by the same non-linear methods mentioned above for data obtained through the other motion tracking systems. However, recent studies evaluated interpersonal coordination by calculating synchronization indexes of specific body parts of participants. For example, Llobera et al. (2016) utilized motion capture suits with 53 reflective markers that enabled the tracking of whole-body movements in dyads who participated in their studies. On the motion capture suits, reflective markers were placed at the levels of joints, such as the neck, shoulders, elbows, wrists, trunk, pelvis, knees, and ankles. Llobera et al. (2016) were interested in analyzing motion data and psychological measures to identify factors related to the subjective sensation of synchrony. To accomplish their purpose, the researchers utilized motion capture suits to record the body movements of dyads who walked spontaneously in a circle and then played a mirror game in two conditions: joint and blind (i.e., with or without visual cues). The researchers assessed spontaneous synchrony during walking by estimating a footstep synchrony index (i.e., a measure of the phase difference between the two participants’ footsteps), expressed as a coordination index ranging from 0 (no coordination) to 1 (simultaneous coordination). In addition, Llobera et al. (2016) evaluated the interpersonal synchronization of each dyad in the mirror game by estimating a hand distance index and a hand speed difference index at specific time points. Their results suggested greater synchrony in the joint condition as well as the prompt emergence of a subjective sense of synchrony as a function of greater degrees of similarity between hand movements of the dyad, negative affect and empathy. They did not find a relation between spontaneous coordination during the circle walk and the subjective sensation of synchrony. Using the same device, procedure and analysis, Preissmann et al. (2016) explored the relation between the subjective sensation of synchrony and the interpersonal synchronization of romantic couples and dyads composed of professional musicians. Their findings showed that musicians synchronized more quickly and effectively than the control group in both tasks; that is, as they spontaneously walked around the circle and while playing the joint version of mirror game. In contrast, these researchers did not find statistically significant differences in the synchronization of the romantic couples versus the control group. Other findings from studies that utilized optical capture systems revealed that speed and coordination between the limbs of couples were independent of the difficulty of the task (Fine and Amazeen, 2011), that synchronization between pairs improved socio-motor improvisation (Gueugnon et al., 2016) and that visual coupling induced spontaneous coordination of participants’ movements (Oullier et al., 2008).

## Psychophysiological and Neurophysiological Methods

Psychophysiological and neurophysiological methods have been utilized to study interpersonal coordination. Whereas psychophysiological studies work with variables such as breathing (McFarland, 2001) and heartbeats (Levenson and Gottman, 1985), brain research investigates the neural activity of people participating in social interactions. The most frequently utilized methods to examine the neurophysiological level of interpersonal coordination are functional magnetic resonance imaging (fMRI) (Jasmin et al., 2016), electroencephalography (EEG) (Lindenberger et al., 2009; Yun et al., 2012), and near-infrared spectroscopy (NIRS) (Cui et al., 2012; Holper et al., 2012).

### Psychophysiological Measures

Heart rate and galvanic skin response are relatively unobtrusive methods that are sometimes well suited to capture bodily dynamics that occur among subjects in different types of interactions at time scales as short as minutes or even seconds. Synchrony of involuntary and automatic psychophysiological responses has been found across a broad range of contexts. For instance, Levenson and Gottman (1985) found evidence of heart rate synchrony between spouses engaged in conversation, and Chatel-Goldman et al. (2014) observed that touching each other increased skin conductance synchrony in dyads. Recently, Mønster et al. (2016) found evidence of skin conductance synchrony among team members during a cooperative task.

Synchrony between heart rhythms is typically computed for dyads using a time series analysis (Feldman et al., 2011) or multivariate recurrence quantification (Mitkidis et al., 2015). Although both analyses compute cross-correlation functions between interbeat intervals of the two members of the dyad, multivariate recurrence quantification can evaluate the relationship among three or more time series and assess their degree of synchrony, which makes it useful in contexts with three or more people interacting together. For example, Feldman et al. (2011) used heart rate to study the dynamics of coordination in mother-infant dyads using electrocardiogram (ECG) signals. Feldman et al. (2011) investigated whether face-to-face interactions would lead to biological synchrony between the mother’s and the infant’s heart rhythms. They analyzed three forms of temporal synchrony: gaze synchrony, affect synchrony, and vocal synchrony. Their results showed that mothers and infants coordinated their heart rhythms within lags less of less than 1 s and that the concordance between maternal and infant heart rhythms, calculated by means of cross-correlation functions, increased significantly during episodes of affect and vocal synchrony compared with non-synchronous moments. This result suggested that human dyads could alter their physiological processes through the coordination of visuo-affective social signals. In a more recent study, Mitkidis et al. (2015) measured participants’ heart rates to study how trust modulates affective bonds between individuals in a group task. They asked pairs of participants to build model cars together using LEGO bricks in four consecutive 10-min sessions. The results showed significantly higher heart rate synchrony, estimated by multivariate recurrence quantification analysis, for participants in the trust condition than for those in the control condition. The authors concluded that changes in physiology and behavior were shaped by the evaluation of other people’s behavior and indicated a trust-building process. Heart synchrony has additionally been found to be related to group cohesion and team trust (Strang et al., 2014).

### Neurophysiological Studies

The study of interactional dynamics from a neurophysiological viewpoint has been made possible by utilizing fMRI, EEG, and NIRS. In this context, the term hyperscanning (Montague et al., 2002; Dumas et al., 2010; Konvalinka and Roepstorff, 2012; Babiloni and Astolfi, 2014) has been applied when any fMRI, EEG or NIRS setup is utilized to simultaneously track two or more brains. The first study of this type was reported by Montague et al. (2002). In their work, two participants were scanned using two different fMRI devices during a simple game in which the receiver had to determine whether the color on his/her screen corresponded to the color originally shared by the sender. They found common activity in the supplementary motor areas of the sender and the receiver.

#### Functional Magnetic Resonance Imaging (fMRI)

Functional magnetic resonance imaging measures the blood oxygen level-dependent response that results from changes in the concentration of paramagnetic deoxyhemoglobin. Due to its relatively high spatial resolution, fMRI is particularly useful for studying interpersonal coordination because it allows comparison of the brain structures involved in any given task and, therefore, their coupling to the other person’s brain. However, due to spatial restrictions imposed by the fMRI setting, interpersonal coordination studies typically make participants believe that they are interacting in real time with another person through some virtual media (Stephens et al., 2010; Earls et al., 2013; Cacioppo et al., 2014). For example, Stephens et al. (2010) independently scanned the brains of two people to study speaker-listener coupling. Native English speakers listened to a recording of a real-life story told in English and in Russian by another participant. The fMRI data of speakers and listeners were later synchronized and compared. In another fMRI study, Cacioppo et al. (2014) told participants that they were exchanging texts with another person in the room; however, they were really interacting with a computer programed to respond synchronously (at the same rhythm) or asynchronously (at a different rhythm) to the player’s tapping.

Synchrony between brains is typically measured by means of coefficients such as coherence, correlation (King-Casas et al., 2005) or Granger-based correlation (Schippers et al., 2010). Typically, this type of analysis measures the activity of two different brains by calculating the correlation or coherence of the same voxels at the same coordinate positions in each brain. Brain activity is measured in either all possible pairs of voxels or in previously defined clusters of voxels and is typically compared using two-sample *t*-tests to ascertain which areas show significantly paired, or coupled, activity. For example, Saito et al. (2010) found correlations in the activity of the right inferior frontal gyrus when participants were interacting with each other in a “joint attention task” inside the scanner under conditions in which they could or could not see the other participant’s eyes on a screen. Correlations in the activity of the right inferior frontal gyrus were not found in trials in which the eyes of the other participant were not shown on the screen, suggesting that this area is related to sharing intention during eye contact. In another real-time interaction study with fMRI, Jasmin et al. (2016) recorded the brain activity of dyads and manipulated whether the pair spoke the same sentence (synchrony condition) or different sentences (not synchronously) and whether the voice was “live” or prerecorded. Synchrony in speech was associated with increased activity in the posterior and anterior auditory fields for both participants, and if the partner was a “live voice,” they observed a lack of suppression of the auditory cortex. FMRI hyperscanning has additionally been utilized in the context of game theory (King-Casas et al., 2005; Tomlin et al., 2006) and in emotion expression/recognition tasks (Anders et al., 2011).

#### Electroencephalography (EEG)

The use of EEG has been extended to study the neuronal dynamics of more than one brain as multiple people perform a given activity (Yun et al., 2012; Konvalinka et al., 2014; Toppi et al., 2016). EEG measures the activation of the dendritic trees of a large population of pyramidal neurons in response to external stimulation. It provides information with high temporal resolution, therefore enabling researchers to study participants’ brains online as they perform a given activity.

In EEG, synchrony between brains is typically measured in frequency domains by estimators such as the principal locking value (Dumas et al., 2010), inter-phase synchronization (Lindenberger et al., 2009) and partial directed coherence (Astolfi et al., 2010), a Granger causality-based measure in the frequency domain (Baccalá and Sameshima, 2001). These estimators seek to assess the relation between frequencies in selected regions of interest in pairs of brains as a measure of inter-brain functional connectivity in the same or different frequency bands. For example, Astolfi et al. (2010) found synchrony in alpha, beta and gamma bands between pairs of participants playing a card game on the same team. Pairs of participants were asked to play against a rival dyad; interestingly, synchrony was not found for the brain activity of the participants on different teams. The results of Astolfi et al. (2010) emphasized that different interactional situations were associated with different patterns of cortical activity. In a study performed by Lindenberger et al. (2009), the brains of eight pairs of guitarists playing a short melody were simultaneously recorded. The results of this study showed that coordinated actions preceded and accompanied inter-brain oscillatory couplings in frequency bands below 20 Hz. In a more recent example, Konvalinka et al. (2014) conducted an EEG hyperscan to explore the neural mechanisms underlying coordinative and complementary behavioral patterns during a tapping task. They required participants (seated with their backs to one another) to tap together coordinately in the experimental condition or to follow a computer metronome in the control condition. When participants interacted with another person but not with the computer metronome, alpha and low-beta oscillations were suppressed over motor and frontal areas. The researchers additionally found asymmetric brain-coupling patterns or complementary patterns of individual brain mechanisms. Specifically, they found frontal alpha suppression, especially for the leader, during the anticipation and execution of the task. Other studies have utilized EEG hyperscanning in paradigms of finger movement and visual contact (Tognoli et al., 2007; Naeem et al., 2012), in the context of the Prisoner’s Dilemma (Fallani et al., 2010; Astolfi et al., 2011), paired with motion tracking devices (Yun et al., 2012), in musical interaction (Babiloni et al., 2011, 2012; Loehr et al., 2013), in a go/no-go task (Sebanz et al., 2006) and when handing other participants objects (Kourtis et al., 2010).

#### Near-Infrared Spectroscopy (NIRS)

Near-infrared spectroscopy measures hemodynamic correlates of neural activity (Cui et al., 2011) by detecting changes in the concentrations of oxy- and deoxyhemoglobin in the brain’s blood supply by emitting two (or more) different light wavelengths that provide continuous quantitative measurements of their variation (Minati et al., 2011).

Synchrony between the activities of two brains is measured by means of wavelet coherence or wavelet transform coherence (Cui et al., 2012; Cheng et al., 2015; Osaka et al., 2015). This technique is used to assess the inter-brain coherence of signals generated by dyads of participants, measuring cross-correlation between two time series as a function of frequency and time (Torrence and Compo, 1998) and revealing which ranges of frequencies are involved when subjects perform a particular task together. For example, Cui et al. (2012) found that inter-brain coherence in the frequency bands between 3.2 and 12.8 s and between 0.3 and 0.08 Hz in the superior frontal cortex increased when participants played side by side at a computer game in which they were required to cooperate but not when they were required to play against each other. These frequencies have additionally been found in cooperative singing (Osaka et al., 2015). In another example, Cheng et al. (2015), using the same task as Cui et al. (2012), found that opposite-gender dyads showed significant brain coherence in frontal regions (frontopolar cortex, orbitofrontal cortex, and left dorsolateral prefrontal cortex), whereas cooperation in same-gender dyads was not associated with such synchronization. More recently, Liu et al. (2016) utilized NIRS to examine the neural bases of cooperation and competition. During their experiment, two participants sat side by side in front of a computer. They played a turn-based game in which they played different roles (e.g., cooperator or competitor with respect to the builder’s actions). In the cooperation condition, researchers found no positive correlations between the frontoparietal activations of the cooperator and the builder. By contrast, in the competition condition, the competitor-builder pairs showed significant inter-brain correlations in the bilateral inferior frontal gyrus and the bilateral inferior parietal lobule. The investigators concluded that activation in the right inferior frontal gyrus was reduced or increased during interaction with a cooperative or competitive partner, respectively. Frontopolar interpersonal neural synchronization in cooperative tasks was additionally found by Nozawa et al. (2016).

## Discussion

Given the variety of methods and growing evidence of coordination at different levels of study, a central task in this area is the coherent theoretical modeling of multiple existing results. Beyond the specificities of the techniques, it is important to highlight that they are describing the same interactional phenomena, though at different levels of analysis. Motion capture data undoubtedly require mathematical treatment that is quite different from that required by EEG hyperscanning and that conducted in automatized video analysis. However, in all of these cases, the phenomenon to be explained is the same: people spontaneously synchronizing their movements while interacting face to face. It is important to prioritize the phenomenon over the methods not only to see the commonalities among results coming from different fields but also to ponder the strengths and limitations of each technique.

### Comparing Techniques: Achievements and Limitations

We have grouped the most commonly utilized techniques for studying IC into three main categories: video analysis, motion capture, and psychobiological techniques. Each is sensitive to different features of interpersonal coordination and has its own limitations. Generally, video analysis has the greatest advantage of capturing a wide variety of interpersonal coordination in natural settings. Finer codifications, such as microanalysis, allow detailed tracking even of subtle movements made by participants, giving a rich account of coordination in interactions. One of the most important achievements of video analysis in the study of interpersonal coordination is the identification, for the first time, of the interactional phenomenon of interpersonal synchrony (Condon and Ogston, 1966). In addition, video techniques established that interpersonal coordination is a basic developmental phenomenon that is detectable from the earliest interactions between neonates and meaningful adults (Condon and Sander, 1974). Video analysis additionally enabled the discovery of the close association between interpersonal coordination and positive affective states such as rapport (Bernieri, 1988), liking and social smoothness (Chartrand and Bargh, 1999). However, this method, particularly microanalysis, is labor intensive and time consuming (Paxton and Dale, 2013a). Recent techniques developed for automating video analysis (e.g., MEA, FDMs, CMA, and digital movement plotting) are faster and considerably less time consuming than microanalysis. Through these automated techniques, previous findings have been confirmed and extended. For example, recent studies have shown that interpersonal coordination increases during interactions characterized as non-conflictive (Paxton and Dale, 2013a,b,c; Tschacher et al., 2014) and interactions between friends (Latif et al., 2014). Additionally, in psychotherapeutic contexts, interpersonal coordination appears to be related to the reduction of negative symptoms (Nagaoka et al., 2006). However, automated video analysis measures only the quantity of movement and omits its direction as well as the purpose and quality of coordinated movements. Furthermore, automated video analysis typically turns out to be insensitive to more subtle coordination patterns. In automatic digital plotting, for example, participants’ hand movements are disregarded because the only source of data is the plotted bi-dimensional coordinates. Therefore, although video analysis potentially captures natural interactions and is certainly inexpensive and versatile, its processing is unavoidably subjective and highly time consuming when conducted by humans, or, in the case of automated analysis, it does not provide information regarding specific or subtle but important movements.

Motion tracking systems identify interpersonal coordination with high spatial and temporal precision, even when IC involves morphologically dissimilar movements (Ellamil et al., 2016; Gueugnon et al., 2016) or micromovements imperceptible through video analysis. Studies using potentiometers, electrogoniometers and magnetic sensors have accurately measured angles and positions adopted by specific body limbs or by objects moved by a person during joint tasks consisting of swinging pendulums, walking, jumping, tapping fingers, rocking in a rocking chair, postural swaying, climbing stairs or playing a software game. Through these motion-tracking technologies, it has been possible to identify the perception of the other as a critical variable for the emergence of interpersonal coordination (Richardson et al., 2005, 2007). Therefore, interpersonal coordination emerges when people have perceptual access (e.g., visual, auditory, or haptic) to their interaction partner (Oullier et al., 2008; Demos et al., 2012; Sofianidis et al., 2012, 2015; Nowicki et al., 2013; Athreya et al., 2014). In addition, motion capture established that interpersonal coordination is more pronounced when people perceive common membership (Miles et al., 2011) and a prosocial disposition (Lumsden et al., 2012) and, conversely, that IC is reduced when the group membership or prosocial mindset is not perceived by the participants (Miles et al., 2010). Due to the utilization of these devices in recent studies, we know that interpersonal coordination is impaired in people diagnosed with mental and developmental disorders (Varlet et al., 2011, 2014b; Marsh et al., 2013). A particularly relevant finding is that interpersonal coordination can be additionally observed spontaneously (i.e., unintentionally) (Richardson et al., 2005, 2007; Del-Monte et al., 2013; Davis, 2016). This fact “is indicative of a larger, more fundamental phenomenon that lies at the foundations of what it means to be social” (Davis, 2016, p. 53). Despite the importance of these findings, it is relevant to note that studies using potentiometers, electrogoniometers, and magnetic sensors have not entirely tracked the bodily dynamics that occur during an interaction. Similarly, other motion tracking systems with infrared sensors and accelerometers that provide interesting opportunities to accurately capture movements of the entire body without mobility restrictions have not been utilized for such a purpose. Instead, studies with infrared markers and accelerometers typically utilize only a few sensors per person, constraining the analysis to the tracking of synchrony between a few specific body parts. Studies with infrared markers and accelerometers have allowed us to ascertain the dynamics of interpersonal coordination in specific contexts that include performance in more complex joint tasks (Varlet et al., 2011; Llobera et al., 2016; Preissmann et al., 2016), in joint musical production (Ragert et al., 2013; Glowinski et al., 2015), in joint dance to a musical rhythm (Toivianen et al., 2010; Burger et al., 2014; Ellamil et al., 2016; Lang et al., 2016; Tarr et al., 2016) and in practicing sports such as rowing (Seifert et al., 2017). Finally, motion-tracking devices have not been utilized to study interpersonal coordination in more natural settings, such as conversational contexts, and have been restricted to highly structured experimental tasks. This restricted range of application limits the understanding of real-life interpersonal coordination.

Physiological and neurophysiological methods record the underlying bodily or neuronal dynamics that occur among participants. Accordingly, psychophysiological techniques have uncovered evidence of heart rate coordination between spouses (Levenson and Gottman, 1985) and mother-infant dyads (Feldman et al., 2011) as well in interactions characterized by mutual trust between participants (Strang et al., 2014). Similarly, coordination of skin conductance has been observed between people performing cooperative work (Strang et al., 2014; Mønster et al., 2016). Studies using NIRS have further examined gender differences in synchronized brain activity (Cheng et al., 2015) and the neural bases of cooperation and competition (Cui et al., 2012; Liu et al., 2016). The main advantage of EEG and NIRS is their capacity to measure brain activity during live interactions among participants, in which subjects can interact together in the same space (Lindenberger et al., 2009; Astolfi et al., 2010; Funane et al., 2011; Cui et al., 2012; Konvalinka et al., 2014; Liu et al., 2016) and in different settings, such as playing cards (Astolfi et al., 2011), playing digital games (Cui et al., 2012; Liu et al., 2016) or playing music (Lindenberger et al., 2009; Babiloni et al., 2011, 2012). Nevertheless, EEG and NIRS have lower spatial resolution than neuroimaging techniques such that they lose sight of the brain areas engaged in interpersonal coordination. Through brain imaging techniques, researchers have discovered that simultaneously performed actions activate specific brain areas, including the right inferior frontal gyrus (Tai et al., 2004; Saito et al., 2010; Yun et al., 2012; Liu et al., 2016). It is relevant to emphasize that these regions coactivate particularly strongly when interactions are of a cooperative nature (Osaka et al., 2015; Liu et al., 2016) and that coactivation is inclined to disappear when interactions imply role asymmetries such as leader-follower (Konvalinka et al., 2014). Despite the capacity of fMRI to explore and compare different brain structures as people engage in interpersonal activities (Saito et al., 2010; Anders et al., 2011; Cacioppo et al., 2014), this method presents important challenges. The main drawback is the restrictive setup required to measure brain activity. The conditions of fMRI, in which participants must remain inside scanners and move as little as possible in a different space from the other participant, call into question the ecological validity of studies that employ this technique.

### Methodological Challenges

Revised evidence suggests that interpersonal coordination is strongly related to the construction and maintenance of a common social and affective space and that it involves the entire bodies of the interacting people. Therefore, IC is a rather complex family of morphological and temporal synchronies that is highly sensitive to socio-affective and contextual variables. Consequently, future research must address at least three methodological challenges to advance the understanding of coordinating phenomena.

The first methodological challenge is the generation of experimental designs that are ecologically more sensitive to the nature of IC. Experimental studies using neurophysiological and motion-tracking methods have revealed that IC favors social bonding because it changes easily in response to various clues indicating the other person’s disposition to interact. When this disposition disappears, IC does as well. This finding suggests that people coordinate their movements as a consequence of a tendency to establish social bonds (Semin and Cacioppo, 2008; Marsh et al., 2009; Cacioppo et al., 2014; Lumsden et al., 2014). Despite their importance, the ecological validity of such findings is questionable because emphasis on accurate measurement has often led to highly structured and unnatural tasks. Researchers have recorded the movements of people trained to perform specific motor actions such as walking, swinging pendulums, rocking in a rocking chair and tapping their fingers. In all these cases, actions were performed together with a social referent such as a prerecorded video from another person performing the same task, a virtual avatar, an alleged online participant that was actually software or another participant trained to execute specific actions. Therefore, studies on IC have not sufficiently considered the social meaning that underlies natural human interactions and have dismissed from the start the chance to experimentally study real-life social interaction.

The second methodological challenge is to measure more reliably the association between IC and socio-affective variables. Evidence from studies using video analysis and motion-tracking methods suggests a strong link between IC and affective involvement. It has been revealed that IC promotes prosocial behaviors and increases feelings such as self-esteem, linking, affiliation, trust and empathy (Chartrand and Bargh, 1999; Wiltermuth and Heath, 2008; Hove and Risen, 2009; Kirschner and Tomasello, 2010; Kupper et al., 2010; Miles et al., 2010; Valdesolo et al., 2010; Vacharkulksemsuk and Fredrickson, 2012; Launay et al., 2013; Marzoli et al., 2013; Cirelli et al., 2014a,b, 2016; Kirschner and Ilari, 2014). People are inclined to sympathize more with and perceive themselves as more similar to those with whom they have coordinated. Additionally, IC has been shown to be enhanced by closeness, positive mood, a prosocial mindset, and affiliative, competitive, cooperative, and recreational interactional contexts (Miles et al., 2010; Valdesolo and Desteno, 2011; Lumsden et al., 2012; Paxton and Dale, 2013a,b,c; Rodrigues and Passos, 2013; Hammal et al., 2014; Latif et al., 2014; Tschacher et al., 2014). These results suggest not only that IC favors social bonding but also that this social connection is fundamentally affective. However, the theoretical relevance of such findings is typically overshadowed by the features of the experimental designs. With some important exceptions (Miles et al., 2010; Paxton and Dale, 2013a; Latif et al., 2014), research in the area often associates IC with participants’ self-reported affective states. Despite numerous biases that may be involved in self-report research, it is still unusual for experimental designs to include the manipulation of affective factors to show their effect on IC. Future research should obtain more reliable measurements regarding the impact of social-affective variables on IC.

Finally, the third methodological challenge consists of the search for methods regarding motion data analysis that approach IC as a complex phenomenon. Research using all of the reviewed methods agrees that IC involves the totality of the organism; therefore, it is not surprising that coordination occurs at multiple levels (psychophysiological, neurophysiological, behavioral, and gestural). However, much of the previous research has focused on capturing and analyzing synchrony of one body limb at a time, and few studies have analyzed multimodal coordination (Louwerse et al., 2012; Paxton and Dale, 2013c). Difficulties concerning mathematical modeling may have limited the possibility of advancing the understanding of IC at multiple levels. However, most experimental studies in this area have described IC in terms of morphological and temporal symmetry. Recent analysis techniques within more naturalistic settings have revealed that IC can adopt morphologically dissimilar patterns at different time scales. For example, it has been shown that newborns and adults simultaneously coordinate their body movements with some speech elements of their interaction partners (Condon and Sander, 1974; Paxton and Dale, 2013c). Similarly, it has been observed that unknown and close people coordinate their movements, either immediately or after a delay, while they chat affiliatively (Paxton and Dale, 2013a; Latif et al., 2014). These findings not only highlight the variety of shapes that IC patterns can adopt but also stress the high sensitivity of IC to the quality of interactions. The challenge is to build experimental designs that approximate real-life interactions while integrating the benefits of high-temporal-precision recordings and a whole-body account of the motion dynamics that occur during real human interaction.

## Author Contributions

All authors of this article satisfy the authorship criteria. CC, ZC, RM, and JP made contributions to the conception of the paper and participated in the writing process by adding substantively relevant content. All authors approved the final version to be published. They all agree to be accountable for all aspects of the text in ensuring that questions related to the accuracy or integrity of any part of the work are appropriately investigated and resolved.

## Conflict of Interest Statement

The authors declare that the research was conducted in the absence of any commercial or financial relationships that could be construed as a potential conflict of interest.
